# Cross-jurisdictional Data Transfer in Health Research: Stakeholder Perceptions on the Role of Law

**DOI:** 10.1007/s41649-024-00283-8

**Published:** 2024-05-11

**Authors:** Hui Yun Chan, Hui Jin Toh, Tamra Lysaght

**Affiliations:** Centre for Biomedical Ethics, Yong Loo Lin School of Medicine, National University of Singapore, Singapore

**Keywords:** Data ethics, Data protection law, Data sharing, GDPR, Health data, International data transfer, Research ethics

## Abstract

Large data-intensive health research programmes benefit from collaboration amongst researchers who may be located in different institutions and international contexts. However, complexities in navigating privacy frameworks and data protection laws across various jurisdictions pose significant challenges to researchers seeking to share or transfer data outside of institutional boundaries. Research on the awareness of data protection and privacy laws amongst stakeholders is limited. Our qualitative study, drawn from a larger project in Singapore, revealed insights into stakeholders’ perceptions of the role of law in cross-national health data research. Stakeholders in our study demonstrated a range of perceptions regarding the role of data protection law in governing the collection and transfer of health data for research. The main criticisms included inadequate legal protection to data and lack of uniformed data protection standards. Despite these criticisms, participants recognised the importance of data protection law in supporting cross-border data transfers and proposed measures to improve perceived limitations of existing laws. These measures include strengthening existing legal framework, establishing contractual agreements and imposing severe punishments for data misuse.

## Background

Multiparty collaborations involving researchers from different institutions and countries are becoming increasingly common (Perrino et al. 2013; UNESCO 2017; Jean-Quartier et al. 2022; Kashyap 2022). This trend is supported by an availability of big data and data linkage technologies. Whilst most international collaborations are occurring between European countries and the US, these partnerships have now broadened to include countries located in the global south and Southeast Asia (Research, Innovation and Enterprise Secretariat 2020; Ministry of Health 2022; Smart Nation Singapore 2023). Singapore, a city-state in Southeast Asia (SEA), invests in local research and development initiatives and international collaborations (National Research Foundation 2023) with UK and Australia in the fields of human health, sustainability, trade, smart nation and digital economy (Kamalski and Plume 2013; White 2021). For these investments to succeed, effective data transfer across institutional and international borders is crucial to ensure that applicable data protection obligations are fulfilled, which contributes to generating trustworthiness in economic and research cooperations.

One of the key factors affecting cross-border data transfer is the presence of regulatory frameworks that researchers need to comply with. Research involving health data, including potentially sensitive health data, is broadly governed by laws relating to human biomedical research, data protection or privacy laws and research ethics guidelines (Scheibner et al. 2020; Xiang and Cai 2021). Although there are regulatory protections to address privacy concerns, health research involving many researchers in different countries has challenged existing privacy laws that may not adequately address risks arising from these activities (Kloss et al. 2018; McGraw and Mandl 2021). Yet, legislation regulating data protection has faced challenges in striking the right balance between protecting individuals from privacy harms without creating excessive burdens on research (Fears et al. 2014) that may generate public benefits.

The range of individuals who have interests in being protected from the harms of privacy and data breaches includes users, contributors and beneficiaries of health and research services. As their data are being used or repurposed for health research that is likely to benefit scientific advancements, whether in the short or long term, their perceptions regarding the role of law and the broader regulatory environment matter. This is because their continued support and willingness to participate in data sharing and transfer activities for health research have significant implications, which in turn affect the continuity of research activities that are beneficial to the population.

Empirical studies on stakeholder awareness of privacy and data protection laws have found mixed results. A study on the General Data Protection Regulation (GDPR) in 28 European countries (Rughinis et al. 2022) broadly suggests that awareness of the law generally correlates with the level of education, occupation and age. Another study comparing data protection awareness between UK and German populations showed a strong emphasis on data protection and security (Pleger et al. 2021). Recent research similarly focused on GDPR awareness amongst European study participants (Vukovic et al. 2022). These studies collectively provide largely European-centric perspectives and are potentially less relevant to address concerns that could arise in collaborations between SEA and European or American counterparts (Trade 2014).

Studies that are focused on regions in Europe, North America, and Oceania have political and cultural attributes that prioritise certain values and interests over others, most notably privacy and personal liberties. These priorities are often reflected broadly in the laws of these regions, including the GDPR and national data protection and privacy laws. Although these values are broadly recognised as important in societies, they may not necessarily be the primary focus in SEA. SEA countries often have different expressions of socio-cultural values and interests that may be understood as more communitarian compared to the individualism often expressed in Anglo-American countries.

Prior research from SEA has explored stakeholder perspectives from Cambodia and Vietnam on matters promoting data sharing across borders such as better understanding of disease epidemiology in public health emergencies (Liverani et al. 2018), healthcare advancements, and future personal health benefits (Kalkman et al. 2019). Obstacles and concerns to cross-border data sharing include differences in national structures and rules that govern data transfer, imbalances in capacities and power (Liverani et al. 2018), apprehension about privacy and security protections (Kalkman et al. 2019), potential breaches of confidentiality and misuse of data in controversial research or through exploitations (Majumder et al. 2016; Kalkman et al. 2019). Whilst providing valuable insights into factors influencing data sharing practises in SEA, these studies do not shed light on the extent of stakeholder awareness regarding the role of law in cross-border data sharing or transfer.

The lack of research on this topic could be due to the lack of resourcing capacity in the region, the perceived importance of data protection and privacy laws in the population or general acceptance of existing inadequate privacy laws. Exploring the level of awareness of the role of law in cross-border data transfer amongst stakeholders is essential to identify gaps in knowledge about the role of data protection law in international data transfer, rectify these gaps and prevent any continuity of undesirable practises.

In the context of Singapore, the Personal Data Protection Act 2012 (PDPA) broadly regulates the collection, use and disclosure of personal data with the aim of protecting the personal data of individuals whilst permitting their use for accepted purposes. This intends to create a balance between protecting data from misuse, sustaining population trust in organisations that collect and use their data and promote a trusted business environment for Singapore. The PDPA provides guidance for international data transfer under Sect. 26, supported further by Personal Data Protection Regulations 2021. The PDPA allows transfer of personal data outside Singapore if specific requirements are met to provide a comparable standard of data protection (Sect. 26). The Regulations provide further requirements on how these conditions could be met.

International collaborations raise questions about how data protection laws apply in cross-border data transfers, which are important considerations for a city-state like Singapore that is heavily invested in scientific developments. For example, under the PDPA, organisations that intend to transfer personal data abroad must ensure that the recipient is legally bound by the law, or under contractual obligations, corporate rules or any other types of agreement that would provide a comparable standard of data protection under the PDPA (e.g. if the recipient of personal data holds specific certification such as the Asia Pacific Economic Cooperation Cross Border Privacy Rules System). It is essential for individuals who are responsible for legal compliance to possess an accurate understanding of the applicable law and being aware of the implications to their work. Given the importance of data protection laws in governing the use, collection and transfer of health data across jurisdictional borders, it is important to understand stakeholders’ perceptions and attitudes towards the role of law in international collaborations involving cross-border data transfer.

## Methods

This qualitative study explores stakeholder perceptions of the law within a health data research ecosystem in Singapore. This study was undertaken as the first stage of mixed methods research aimed at developing an ethical code to guide the collection, use and transfer of potentially sensitive health data in Singapore for researchers at the Future Health Technologies (FHT) programme (Lysaght et al. 2023). The FHT is an international research collaboration between the Singapore National Research Foundation and ETH-Zurich (The Swiss Federal Institute of Technology), which established the Singapore-ETH Centre (SEC) to improve health through digital health technologies.

Through pre-existing networks, we identified five groups of stakeholders representing data contributors, data generators, data resources, data facilitators and professional data users. From April to June 2022, we conducted semi-structured interviews with these stakeholders who were invited to participate via email. Written informed consent was obtained prior to the interview. Participants were given SGD$42 token of appreciation. Ethics approval for the protocol was obtained from the Institutional Review Board of the National University of Singapore (NUS-IRB-2022–46).

Interviews were conducted either in person or online according to participant preferences. The interview guide (see Annex 1) was developed with reference to findings from the internal mapping exercise conducted with FHT researchers and our prior studies (Lysaght et al. 2020, 2021; Ong et al. 2021; Ballantyne et al. 2022). Topics are related to (i) health data sensitivity, (ii) international data sharing for research and (iii) regulation and governance. The topic guide was piloted with two senior researchers with experience in qualitative interviews and in health law and ethics. Prompts were used in the interviews to encourage the participants to consider the questions further and elaborate on their responses if necessary. The interviewers took field notes during the interviews and summarised their reflections with each other after each interview.

Interviews were audio-recorded and transcribed ad verbatim. Transcripts were pseudo-anonymised being transferred to NVivo 12 (QSR International) for analysis. Data were coded for qualitative thematic analysis (Miles and Huberman 1994) by the two study team members (HYC and HJT) independently, and codes were compared to identify any discrepancies. Meetings were then held between both members to resolve the discrepancies. The analysis occurred alongside the collection of interviews, and we stopped recruiting after reaching thematic saturation. Thematic saturation was reached when no new relevant themes and their relation to each other were identified (Corbin and Strauss 2014). The coding tree from our previous studies (Lysaght et al. 2020; Ong et al. 2021) was applied to the analysis and was built as new concepts and themes emerged. Codes were merged into broader themes and subthemes and presented to a senior researcher (TL) over several meetings. During these meetings, the senior researcher (TL) reviewed the interview data to ensure consistency in analysis across various themes. We used the Consolidated Criteria for Reporting Qualitative Research (COREQ) guidelines to report on the results.

## Results

Of the 37 potential stakeholders who were invited, 28 agreed to participate, 7 declined due to a lack of availability, and the remaining 2 did not respond. We recruited 28 stakeholders from the five groups: data contributors (*N* = 5), data generators (*N* = 8), data resources (*N* = 4), data facilitators (*N* = 6), and professional data users (*N* = 5). Data contributors were represented by consumer or patient advocates and drawn from local patient support networks. Data generators included researchers working across the FHT programme in both Singapore and Switzerland. Data sources were comprised of hospital data controllers or data access guardians who have authority to grant or refuse permissions to requests to access data in their control. Data facilitators were regulators, industry stakeholders and in relation to the FHT programme, those in charge of establishing IT infrastructure and data security measures. Professional data users comprised of clinicians and industry partners of the FHT programme. Of the 28 interviews, 15 were held online, and the average duration of each interview was 82 min.

The main themes that emerged were related to adequacy perceptions towards local data protection law in safeguarding data privacy in cross-border data transfer and proposals for solutions to strengthen data protection law. Participants expressed a range of views regarding the PDPA, from the law being seen as adequate in some respects, such as establishing minimum standards of compliance, to expressions of ambiguity in terms of scope, coverage and definitions, and ineffectiveness in protecting consumers from service providers who collect vast amounts of unnecessary information that would have been contrary to the GDPR. More participants expressed inadequacies of current data protection law in protecting transferred or shared data. The views were classified under (a) adequacy of existing laws, (b) weaknesses of current laws and (c) limited awareness and accurate understanding of data protection laws.

Participants’ perceptions of the role of law were made within a broader context pertaining to collecting potentially sensitive information for health research and their experience with international data sharing for research through collaborations with local and overseas research institutions. Their attitudes towards the role of data protection laws, particularly the PDPA, are therefore expressed in connection with a perceived ease or difficulty with navigating the legal requirements and the strength and weaknesses of existing local data protection laws in protecting potentially sensitive information in health research.

Additionally, the views from participants that revealed a spectrum of adequacy perceptions towards local data protection law in safeguarding data privacy during cross-border transfer were made in response to broader probes related to the reputation of particular jurisdictions in data protection and the adequacy of laws existing in those jurisdictions in protecting personal data. Responses towards strengthening weaknesses of the PDPA are likely to result from an interest in improving transparency and accountability in the legal framework for collecting and transferring health information.

(i) Spectrums of adequacy perceptions towards local data protection law in safeguarding data privacy in cross-border data transfer
(ii) Existing data protection laws are adequate in protecting shared data for research

Some participants from the professional data users’ group were satisfied with current data protection laws in Singapore in protecting shared data. Participants who expressed that the PDPA provides sufficient protection to data referred to the ease of conducting research under the PDPA. According to these participants, the PDPA has established a minimum standard of protection, resulting in a right balance between providing guidance for legal compliance and enabling the conduct of research.

‘…when an institution is dealing with data in Singapore, they are held accountable according to the Singaporeans regulations and law. Same when you do something in Switzerland, if you hold data, something goes wrong, you’re held accountable according to these laws’.—P17

(b) Data protection laws are reactive, vague and provide limited protection and enforcement

Participants from primarily data generators and resources groups who criticised the PDPA expressed that its developments often lagged behind technological advancements, resulting in the inability to proactively prevent data breaches. Participants attributed the law’s reactivity to its complaint-based approach, citing a lack of awareness of the law in the population, hampering the process of raising complaints and resulting in weak legal enforcement. Additionally, these participants expressed their doubts about the PDPA’s clarity in definitional coverage and scope relating to identifiable and non-identifiable data, which permitted varying interpretations on the identifiability of the data, potentially leading to exploitation risks.

‘Our enforcement model is based purely on complaints. That means if there’s a data breach, and we complain about it, then they will be fined la. So the law is satisfactory for, is sufficient for people who are more “gung ho” (determined and enthusiastic in Hokkien), and who are willing to take risks, and the law is sufficient for people who are risk averse, because it does not, it does not give you a legal standard. It basically just says make sure there is no patient data that is re-identifiable. But it won’t tell you what that means exactly’.—P6

‘… So I do think that a certain level, it affords a minimum level of protection for individuals, and if you read the legislation carefully right, it’s actually quite ambiguous in several areas, right’.—P7

Some participants from the data generator and data facilitator groups expressed that they were often required to provide more information than necessary in Singapore compared to other countries when signing up for services. This approach could potentially widen the opportunities for collected data to be unlawfully accessed. The lack of legal enforcement of the law was also cited as contributing to its weakness.

*I’m getting spam calls spam texts all the time. And I’m wondering, how is that possible. And so makes me question whether, you know, your information is really protected and unregulated, you know. A lot of the time as well, you know, if you want to, if you want to find out about a service, you've got to provide all your personal details and not quite clear why. So that people give more information that they might not necessarily need. And that would be against GDPR. So I wonder, then whether the data protection policy here is sufficient enough.—P2*

Participants expressed various views about the PDPA’s adequacy in protecting shared or transferred data. Amongst the participants who claimed that the PDPA does not provide sufficient data protection, they described the PDPA and related guidelines as lacking in clarity. However, participants who have prior experience collaborating with overseas partners and have transferred anonymised data did not consider the PDPA as problematic, as long as the data were not personally identifiable.

The researchers interviewed generally did not think it would be possible to reidentify an individual where anonymised data were used, although one participant cautioned that data can never be truly anonymised. When probed further, the participant responded that reverse engineering techniques could result in re-identification of individuals because of the existence of another ‘key’ to access identifiable information.

(c) Limited awareness and accurate understanding about data protection and the PDPA

When asked about how data research is regulated in Singapore, all participants were aware of the PDPA. However, they demonstrated a range of awareness when probed about specific knowledge regarding the PDPA. Most participants were unable to articulate the application of the PDPA when probed. Amongst the minority from data resources group who expressed some familiarity with the PDPA, they expressed that Singaporeans generally lack awareness about the PDPA or have limited knowledge about the law.

‘So when I work with government agencies, or maybe even private sector clients, they might not be as knowledgeable about all these policies. Or maybe even government sector, they might not know it until some person from the compliance department come and say, Oh, actually, you need to do that’.—P25

‘I know some basics (of the data protection law), whichever was told in SEC (Singapore-ETH Centre), upon joining in SEC how data is shared and what is the protocol. I think it is PDPA compliance. So those basic things I know but I don’t really know the fine print, if you ask me to dictate some term I will not know’.—P4

None of the participants mentioned the Public Sector (Governance) Act 2018, which applies to government sectors in matters relating to data protection by the public sector in Singapore. This law provides a framework for the government in matters relating to data sharing and disclosure of personal information for the purpose of delivering public services to the population. Some participants’ misconceptions about the PDPA emerged in several aspects. Examples ranged from the view that data transfer is not permissible at all under the PDPA, to the PDPA as a routine training course that formed part of employment onboarding processes without necessarily appreciating its importance and a lack of familiarity with PDPC decisions that applied the principles of the PDPA.

‘I believe our understanding has been that in this project, no data can leave Singapore basically. That is we’re working under that assumption, at least. Or at least that will require some permissions, I suppose… Otherwise, we would probably have just processed all the data on the computer clusters that we have at ETH, but we’re going through this painful process of setting up our own clusters of SECure servers at the NUS site, in order to be able to process all data within it in Singapore…’*—*P23

Section 26 of the PDPA provides for legal mechanisms to transfer data outside of Singapore; however, none of the participants expressed an awareness of this legal provision or mentioned the possibility of data transfer under the PDPA. A few participants from data generators group expressed that researchers are ‘overregulated’ compared to the private sectors under the PDPA due to the necessity to comply with data protection laws and institutional review boards research requirements. These participants who are researchers and have sought ethics approval for research projects expressed that private commercial companies appeared to have greater ‘flexibility’ in gathering consumer data without burdensome regulatory oversight, unlike researchers. This perception could have arisen from the challenges researchers face in fulfilling administrative requirements when gathering research data from individuals. Research involving human subjects could include highly sensitive information, thus requiring greater oversight from ethics committee compared to private companies that are broadly collecting personal information in return for providing services. This difference in perception could have resulted in additional compliance ‘burdens’ being perceived as limitations of the law in supporting cross-jurisdictional data transfer.

‘And it should be the other area where it doesn’t seem to be regulated, that’s the area for me that they (the regulators) should be shutting down on. These, you know, these analytical companies that just take your information and use it, I find that quite dangerous, you know, companies getting huge advantages, economically as well. Whereas people doing research are typically extremely ethical about what they do. They’re very careful, they adhere to regulation, you know, and they look after the data properly, right? They don’t abuse it, they’re not making a profit out of it, there’s no conflict of interest’.—P21

(b) Proposed solutions to strengthen the law

Participants were asked to identify potential non-legal measures that would allow health information sharing for research. In response, most of them identified the government as *the* responsible body to ensure that proper data collection and transfer procedures are in place. Some participants favoured government involvement in international research collaborations involving data transfer, such as forming public–private partnerships. Additionally, participants proposed that laws should be enforced with stronger accountability in response to perceived insufficiencies of the PDPA through various means such as contractual agreements, audits and selecting reputable research partners. Another suggested measure was transferring data *only* with a clear, necessary purpose (e.g. lack of local resources requiring overseas expertise) and strong public benefit.

‘So the use of the data is for the benefit of the project, or the service, or the individual or the society or whatever. And that remains the same and how, like it’s been used in a good way, right? But then if you’ve got all these data, and you’re like, oh, I can give this to someone else who can then go in and sell it for some other purpose to make a profit or to do something else with it, then that's not okay to me’.—P2

When asked about measures to resolve weaknesses of the law, participants proposed heavier fines for data misuse and breach, proactive monitoring to ensure adherence to data protection measures and penalties for failure to comply with protective measures. Some participants recommended strengthening current data protection laws and applicable policies. They referred to specific privacy-related standards such as Health Insurance Portability and Accountability Act (HIPAA) to improve the limitations of the PDPA.

‘International data sharing. I think what has always been an issue is the cross recognition, right? Of course, we have our PDPA. Europe has the GDPR and the US has the HIPAA. How the standards talk to each other? I think that’s why it’s a bit challenging in the Data Protection world to find an equivalence because it really depends on your social, what your society and culture beliefs about why it is sensitive’.—P26

One of the reasons cited was the benefit of referring to examples to assist people in implementing the PDPA concerning data collection and transfer, such as expectations of security measures. Examples cited include how to ensure data are secured (e.g. securely locked in metal cabinets), which would assure companies that they are properly securing their data. A further suggestion was creating a special commission to guide implementers in interpreting the law to assist with compliance.

Additionally, participants expressed complications in assessing the risks arising from sharing and transferring data and potential benefits from such activities. Although participants expressed concerns about risks from sharing and transfer (e.g. data breaches or security lapses), they reasoned that these risks were inevitable. What was important to them was the response to breaches. This reaction seemed to follow from their trust in the government as a trustworthy actor despite previous incidents of breaches. Participants who favoured this view expressed the need to strike a balance between the risks from transferring data overseas and benefits from research using transferred data.

‘From the healthcare angle, there is definite benefits to access the use of data. And even though in the past there are cases where data are abused with malicious intent, it is still acceptable risk’.—P20

Some researcher participants expressed the lack of standardised global data protection guidelines as complicating collaborations with international partners and challenging in implementing accountability when data breaches occur as the PDPA is not recognised outside of Singapore. Despite this view, they recognised the challenge in standardising laws and regulations on data privacy and protection across countries given differences in socio-cultural and political contexts.

Most participants expressed support for the use of legally binding contractual agreements (e.g. research collaboration agreements) with overseas partners to address accountability concerns. These participants explained that specific terms and conditions such as purpose of use, references to existing data protection policies and penalties for non-compliance could be included in these agreements. They added that contractual agreements provide the opportunity for parties to demonstrate their intention to comply with the agreement in the data sharing and transfer relationship.

## Discussion

This study is aimed at exploring the perceptions stakeholders have towards data protection laws and their impacts on research in Singapore. Previous studies have explored the perceptions of stakeholders on the role of data protection or the extent of awareness of privacy laws relating to health research. A study exploring stakeholder perspectives on the enablers and barriers of the GDPR for cross-border health data sharing in Europe (Vukovic et al. 2022) revealed positive perceptions towards the GDPR in facilitating the secondary use of health data in relation to user rights over their data, including existing laws governing data privacy and sharing. Barriers include perceived lengthy times for completing the process of data access, an increase in workload and differences with domestic legislations and differing interpretations to data access. These aspects were not reflected in our study, most likely due to differences between GDPR and PDPA requirements relating to how potentially sensitive data are treated under these rules.

### Challenges with Implementing the PDPA in relation to Data Protection

In terms of stakeholders’ attitudes regarding how the PDPA has been implemented, there were some similarities with prior studies investigating public expectations about data protection laws. A study exploring public preferences for using identifiable data without consent in the light of differences in data protection laws in the US showed the need to align these laws with public preferences that are supportive of data use for research and public health. The study further revealed that researchers felt unreasonably burdened by uncertainties in implementation of laws governing the sharing of health data, as they were frequently ill-equipped to deal with this aspect of their data sharing activities (Genevieve et al. 2021).

Our analysis indicated similar attitudes amongst some participants, which revealed some uncertainties regarding the application of the law and an absence of alignments between participants’ expectation of the PDPA and its implementation relating to the scope of application and enforcement. This could have contributed to greater expressions of dissatisfactions about the efficacy of the PDPA. Participants from data generators group were more likely to be concerned about meeting the legal requirements surrounding data transfer, whilst data contributors were interested in understanding how their data could be protected under the law and implications arising from any likely harms associated with sharing these data for research. The former could be due to familiarity with seeking ethical approvals in their research and the necessity of ensuring compliance with current laws.

The divergences in priorities could potentially translate to distinct expectations regarding the law. It can be postulated that the views expressed by our participants were informed by their backgrounds ranging from patient support networks, scientists researching in digital health technologies, data controllers in hospitals, regulators, clinicians and industry representatives. The multidisciplinary nature of their perspectives could thus potentially influence their expectations about the PDPA and affect the way they apply the requirements of the PDPA or related privacy laws in their professional work.

The PDPA was initially introduced to implement the Do-Not-Call Registry (PDPC 2013), but has since developed to accommodate growing privacy concerns, evidenced by the publication of guidance notes and decisions by the Personal Data Protection Commission (PDPC). However, our participants appeared unaware of enforcement decisions against companies that breached PDPA obligations (PDPC 2023b) or the availability of complaints for data breaches relating to data protection, do-not-call, unlicensed loans or online gambling and government data incident reporting platform (PDPC 2023c). Although the publication of these decisions is intended to demonstrate that PDPA compliance is vital, it is unknown to what extent the populations are aware of these decisions and the implications to their lives. These decisions however assist commercial companies in meeting their obligations under the PDPA in relation to data collection, transfer and protection standards.

The participants’ dissatisfaction regarding the fines for PDPA breaches could be attributed to the focus on economic growth and innovations from international collaborations within Singapore, where data protection rules that are considered as obstructing health research might deter overseas institutions from collaborating with Singapore (BSA 2020). As a result, an ‘acceptable’ trade-off is a perceived ‘weaker’ protection level (or minimum protection standards) but meets the minimum protection standards that are conducive for commercial companies or scientific research. Policy makers in Singapore therefore need to navigate this trade-off carefully by considering economic growth, international collaborations, and the importance of safeguarding individuals’ privacy.

The limitations of the PDPA are seen by participants as an obstacle to genuine data protection. It could be valuable for public outreach activities (PDPC 2023a) to widen PDPA awareness, including educating the public about ways to protect their personal data from being misused, consistent with one of the functions of the PDPC. The Personal Data Protection Commission (PDPC) plays an important role in a range of matters related to data protection, such as promoting awareness of data protection in Singapore, providing advisory and consultancy services relevant to data protection, enforcing the PDPA, conducting educational activities relating to data protection and managing cooperation with other organisations in areas of data protection. Perhaps this approach could assist in realigning public expectations about the PDPA and its implementation and to enable the public to acquire a better understanding of the PDPA and the role of the PDPC.

A majority of the participants who expressed dissatisfaction with the PDPA and its implementation governing the collection, use and transfer of potentially sensitive information remain interested in its role and proposed measures to strengthen the law. The recommendation to introduce higher fines for data breaches is consistent with recent amendments to the PDPA (Amendment) Act 2020. The change to the law under Sect. 48J(3) of the PDPA provides a higher imposition of penalties for data breaches from $1 million to 10% of the organisation’s annual turnover for organisations with over $10 million annual turnover and $1 million for all other cases.

Further, Sect. 48J(6) of the PDPA enables the PDPC to consider a range of matters in determining the amount of financial penalty such as the proportionality and efficacy of the financial penalties, or any previous non-compliance and the nature, gravity and duration of non-compliance. These amendments could be better received by the stakeholders, as it allows for fair consideration of individual cases rather than a one-size-fits-all approach. The amendments also reflected a recognition that data breaches are harmful to affected individuals and the penalties are intended to deter future breaches or seen to be acting justly for the harm caused to the affected people. Such a recognition may resonate better with stakeholders as it fosters a perception that the law is responsive to the concerns and rights of individuals whose data are compromised.

Overall, these amendments are likely to change participants’ perception of PDPA enforcement by positioning it as a more formidable and responsive framework. The emphasis on substantial penalties for non-compliance suggests a commitment to ensuring greater protection for individuals, which could instil confidence in the effectiveness of the PDPA in safeguarding personal data. However, the true impact will depend on how effectively these measures are implemented and enforced in practise.

Trade-off considerations between privacy and research benefits from cross-border data transfer appeared to be a persistent challenge (Sarabdeen and Moonesar 2018; Schmit et al. 2021). A study investigating stakeholder perspectives about the Protection of Personal Information Act 2013 in South Africa (Staunton et al. 2021) showed tensions between strengthening data protection and using personal data for care delivery and research, raising concerns that need to be addressed through increasing an understanding of the law in data protection, improving accountability and transparency in data use. Tensions that arose in the study reflected similar trade-off considerations as expressed by our study participants. This aspect further highlighted the difficulty in striking a balance between ensuring appropriate privacy protection for data that are subject to cross-border transfers and enabling international research to continue.

### Comparisons between the PDPA, the GDPR and the HIPAA as Data Protection Standards

The influence of UK-US privacy frameworks and data protection approaches appeared to be high in some of our participants. This perception seemed to comport with studies on the GDPR, which was said to provide better data protection schemes compared to national laws (Gabel and Hickman 2019; Robichaud 2020; Pop 2023). This could be due to a greater uniformity within the European region in implementation and enforcement of the laws, supported by guidance to deal with any inconsistencies in application (Gabel and Hickman 2019; Saunders and Reifman 2021). References to European-centric privacy framework rather than domestic regional standards, such as the Asia Pacific regional standard to data protection that was introduced under the APEC Privacy Framework in 2016 (APEC Privacy Framework 2005), could be attributed to past or existing collaborations with research partners who are primarily located in European countries or the USA, thus necessitating compliance with these data protection standards.

Additionally, although comparisons were frequently made between the PDPA and the GDPR, the views varied depending upon participants’ experiences in applying PDPA and the context in which they are implemented. For instance, some participants from data generator group demonstrated inaccurate understanding about the PDPA whilst others had very limited awareness of the content other than the existence of the law. A lack of clear articulation of how the law is applied amongst some participants could have arisen from an environment where legal compliance with data protection is delegated to legal departments. The lack of knowledge likely influenced their views about how data are collected and transferred under the PDPA and the adequacy of the protection to their data. A report (National Board of Trade 2014) describing the knowledge of data protection regulations amongst companies in Sweden revealed a similar variation of awareness of the local data protection law. The report noted that whilst the surveyed companies were aware of the law, their interpretations differed resulting in a lack of accurate comprehension of how the rules implicate their business operations. This in turn could affect the safety of their customers’ personal data whilst operating their businesses.

Although several participants perceived the HIPAA’s articulation of privacy standards as more helpful compared to the PDPA, it is unclear whether they are aware of the lack of the HIPAA’s adequacy privacy standard (similar to the PDPA, which is not considered to have adequacy equivalence). Currently, only a limited number of jurisdictions are assessed as possessing privacy adequacy standards corresponding to GDPR standards (European Commission n.d), such as Switzerland, New Zealand, the Republic of Korea and the US (but limited to commercial parties in the EU-US Data Privacy Framework). Whilst a survey in 2022 revealed an increase in awareness of data privacy laws in Singaporean consumers (SMEhorizon 2022), the findings were self-reported and they might have misconceptions about data privacy laws, similar to our participants.

Important implications for Singapore include the need for targeted awareness campaigns to address potential misconceptions and improve understanding of data privacy laws. This could include educational initiatives, communication strategies, and potentially regulatory measures to ensure that individuals and businesses are accurately informed about their rights and responsibilities under data protection laws. In addition, considering the global scope of data transfers and privacy standards, Singapore could consider exploring opportunities to better align its regulations with international frameworks to improve adequacy equivalence.

Our participants’ preference for contractual agreements in establishing obligations and expectations between research collaborators is aligned with current practises for cross-border research collaborations. Researchers perceived these agreements as flexible, adaptive and secure to protect the interests of the parties because they can negotiate the terms and conditions (Hallinan et al. 2021). Specific contractual terms covering data access or sharing may be included in separate data access/use agreements as safeguards for data sharing obligations (Mazor et al. 2017; Kalkman et al. 2019). Whilst these agreements are perceived as a form of accountability when data breaches occur, their efficacy may be limited by trans-territorial applications of the law relating to enforceability. There are other practical and legal limitations to ensuring accountability of third parties in the event of a breach such as determining appropriate compensations or responsible parties (Van Asbroeck 2019; OCED 2023). These limitations are consistent with participants’ expressed doubts about the extent of actual compliance with agreements.

## Limitations and Future Research

This study has several limitations. First, most of the participants representing researchers from different nationalities may have limited knowledge and awareness of local data transfer or data protection laws. References to data protection laws are thus often made to more well-known frameworks such as the GDPR and the HIPAA. However, a group of participants comprising local expertise in data protection has provided a local understanding of their work experience in implementing data protection and transfer requirements.

Participants have a varied understanding and knowledge of the PDPA which presented a spectrum of views regarding its adequacy in data protection and data transfer for research. Their understanding of the law is highly influenced by their personal and work experience, which is generally accepted as not representative of the population. Further studies with a bigger sample of similar stakeholders may be able to validate this point. Nonetheless, the interviews have yielded rich responses from study participants that enabled the research team to have a clearer understanding of law-related awareness and perceptions in cross-jurisdictional health data transfers.

Additionally, as this study explored perspectives in Singapore with its unique culture and political environment, the results may not be generalisable to other countries but is sufficiently valuable to prompt discussions and further research on the relevance of data protection laws in facilitating cross-border data transfers. Further research could include evaluating the practical impact of the PDPA on facilitating or hindering cross-border health data transfers. In particular, it could involve assessing the effectiveness of the law in achieving its intended objectives, as well as identifying any challenges or gaps that may exist in the current regulatory framework. Another potential area of future research would be to conduct longitudinal studies to measure changes in awareness, perceptions and compliance with the PDPA over time. This can help policymakers anticipate and address emerging issues as the digital landscape and technologies evolve.

## Conclusion

Our study showed that stakeholders perceived data protection laws as playing an important role in cross-border data transfer despite some inadequacies. Criticisms regarding the PDPA are often directed at the lack of clarity in its scope and definitions for researchers and the perceived lack of enforcement to penalise data misuse or breaches. However, some stakeholders’ perceptions of the law were influenced by an inaccurate understanding and misconception about the law. It is therefore important to increase awareness on data protection laws for stakeholders who are working in the health research ecosystems and the public generally. Attention could be drawn to highlighting the efforts of the PDPC in enforcing data protection laws through publication of decisions regarding data protection breaches by companies in Singapore. Participants advocated the need for strengthening enforcement on existing data protection laws, ensuring accountability of overseas collaborators through legally binding contractual agreements and imposing greater punishments for contravening contractual obligations relevant to data protection.

## Data Availability

The data that support the findings of this study are available on request from the authors.

## Annex 1

**Interview Guide**

1. **Introduction and build rapport**
  - Discuss participants’ professional background and experience with data research, if any
2. **Health data sensitivity**
  - Explore what types of health-related information participants would consider as ‘sensitive’ (e.g. medical records, clinical diagnoses, hospital stays, mental health status, pathology test results, and whole genome sequences) and contrast with potentially sensitive non-health information (e.g. financial/banking statements) and health data collected from mobile phone apps, wearable devices, IoTs, contact tracing, etc.
  - Probe on what makes certain types of health information ‘sensitive’ or not, if there are different levels of sensitivity, and if there should be greater levels of restrictions on who can access such information. If so, what sorts of restrictions?
3. **International data sharing for research**
  - Explore participants’ views about sensitive and non-sensitive health information being stored on cloud servers that are accessible to international collaborators at publicly funded research institutions/universities overseas and contrast with industry collaborators having access to the data or if the data are sold to commercial company that is developing medical products
  - Probe on whether the purpose of the research matters, or the country or region where the collaborators are located (e.g. the USA or EU country vs. China or North Korea), the prestige or visibility of the university/institution or company
  - Explore the conditions that participants would allow sensitive health information to be stored in Singapore and shared with international collaborators (e.g. IRB review, public benefit, removing personal identifiers, and informed consent)
4. **Regulation and governance**
  - Establish how much participants know about how data research is regulated in Singapore and whether they believe the regulations are sufficient for sensitive health information, however that is understood
  - Probe on matters of consent and understanding about the trade-offs in requiring informed consent every time data are shared/accessed with the added costs of recontacting participants multiple times and risks of reidentification
  - Identify any non-legal measures that could be put in place to allow sharing of health information for research (both locally and overseas) without needing to reconsent each time
  - Probe for the contours of what types of research is acceptable (e.g. disease specific and product development) or unacceptable (e.g. heritable genome editing and human cloning), and the sorts of public benefits that can be expected (or not) from research with patient health information
  - Probe on matters of transparency and accountability and what measures are reasonable and feasible for researchers to access patient health information without obtaining informed consent each time
